# A Novel High-Performance Beam-Supported Membrane Structure with Enhanced Design Flexibility for Partial Discharge Detection

**DOI:** 10.3390/s17030593

**Published:** 2017-03-15

**Authors:** Chenzhao Fu, Wenrong Si, Haoyong Li, Delin Li, Peng Yuan, Yiting Yu

**Affiliations:** 1 State Grid Shanghai Electric Power Research Institute, Shanghai 200437, China; 13512111246@139.com (C.F.); siwenrong@126.com (W.S.); 2 Key Laboratory of Micro/Nano Systems for Aerospace (Ministry of Education), Northwestern Polytechnical University, Xi’an 710072, China; lyhaoyong@mail.nwpu.edu.cn (H.L.); delinli@mail.nwpu.edu.cn (D.L.); 3 Key Laboratory of Micro- and Nano-Electro-Mechanical Systems of Shaanxi Province, Northwestern Polytechnical University, Xi’an 710072, China; 4 Xi’an Maorong Power Equipment Co., Ltd., Xi’an 710048, China; y5anpeng@126.com

**Keywords:** optical fiber sensors, Fabry-Perot interferometer, beam-supported membrane, partial discharges (PDs)

## Abstract

A novel beam-supported membrane (BSM) structure for the fiber optic extrinsic Fabry-Perot interferometer (EFPI) sensors showing an enhanced performance and an improved resistance to the temperature change was proposed for detecting partial discharges (PDs). The fundamental frequency, sensitivity, linear range, and flatness of the BSM structure were investigated by employing the finite element simulations. Compared with the intact membrane (IM) structure commonly used by EFPI sensors, BSM structure provides extra geometrical parameters to define the fundamental frequency when the diameter of the whole membrane and its thickness is determined, resulting in an enhanced design flexibility of the sensor structure. According to the simulation results, it is noted that BSM structure not only shows a much higher sensitivity (increased by almost four times for some cases), and a wider working range of fundamental frequency to choose, but also an improved linear range, making the system development much easier. In addition, BSM structure presents a better flatness than its IM counterpart, providing an increased signal-to-noise ratio (SNR). A further improvement of performance is thought to be possible with a step-forward structural optimization. The BSM structure shows a great potential to design the EFPI sensors, as well as others for detecting the acoustic signals.

## 1. Introduction

The occurrence of partial discharges (PDs) within power transformers is prone to cause insulation breakdown, which can lead to catastrophic accidents including casualties and a huge loss in economy [1]. PDs detection provides an important way to monitor the working condition of power transformers and prevent the potential insulation breakdown in advance [2].

Generally, PDs will generate pulse currents, accompanied by the gas, light, and ultrasonic byproducts that can be utilized for characterizing the PDs. In practice, the PDs detection mainly includes the pulse current method, as well as the chemical, optical, and ultrasonic detection methods [3,4,5]. Usually, the pulse current method is an easy way to detect the strength of discharging current and determine the specific type of PDs. However, it is difficult to ascertain the location of PDs and is not suitable to apply for the transformers in operation because of low resistance to the electromagnetic interference (EMI) [6]. The chemical detection method shows the advantage of being immune to EMI and stable to work on line. Nevertheless, it is poor for recognizing the type and location of PDs, and also presents low sensitivity and responsive speed, which are essential for detecting the PDs. A key problem of the optical detection method is the exceedingly weak optical signal that is hard to detect. As a result, it is not often adopted [7,8]. More recently, fiber optic extrinsic Fabry-Perot interferometer (EFPI) sensors utilizing the ultrasonic signals are proposed for detecting the PDs in the transformers, because the EFPI sensors have several inherent advantages. These advantages include small size, light weight, high frequency response, electrical insulation, and immunity to EMI noise, all of which make the optical fiber EFPI sensors meet the most conditions necessary to detect the PDs. In the meantime, the EFPI sensors function reliably inside the transformers, even deep within the transformers to pick up the clean PD-induced acoustic signals; they are also safe and easy to install [9,10,11]. However, the sensitivity of EFPI sensors is relatively low compared with the other detection methods, making the detection of weak PD signals a difficult task [12].

In this research, a novel beam-supported membrane (BSM) structure with an enhanced design flexibility for the EFPI sensors is proposed and investigated based on the theoretical analysis and numerical simulation of finite element method (FEM), showing an improved resistance to the temperature change, a much higher sensitivity, a larger linear range, and a better flatness than the commonly used intact membrane (IM) structure. The demonstrated idea for designing the EFPI sensor system is potential for monitoring the weak signals induced by the PD activities in power transformers, as well as other acoustic events.

## 2. Sensor Design

**Figure 1:**
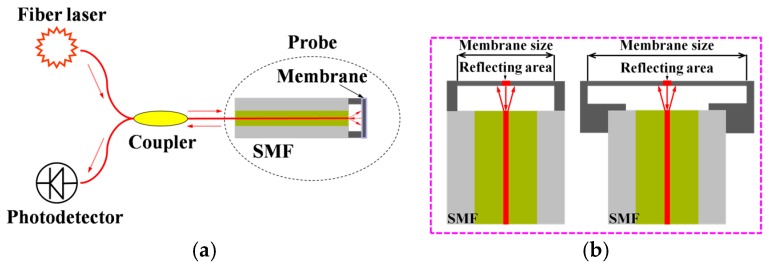
The schematics of (**a**) the whole fiber optic extrinsic Fabry-Perot interferometer (EFPI) sensor system; and (**b**) two assembly manners for the Fabry-Perot cavity with different membrane sizes.

The schematic of the whole fiber optic EFPI sensor system working with the intensity interrogation is shown in Figure 1a, which is composed of a sensor probe, a fiber optic coupler, a fiber laser, and a photodetector. Furthermore, the sensor probe mainly includes a single-mode fiber (SMF) and a membrane structure to sense the acoustic signals, which will be eventually sealed to avoid contact with the oil in the transformer. In this system, the light emitted by the laser passes through the fiber optic coupler and the SMF to reach the reflective membrane structure, between which and the end surface of SMF forms a Fabry–Perot interferometer; two possible assembly manners for the Fabry-Perot cavity with different membrane sizes are shown in Figure 1b. In the sensor probe, the interference light retransmits through the SMF and the fiber optic coupler to get to the photodetector. When the PDs occur in the transformer, the generated acoustic vibrations excite the membrane structure and cause a structural deformation, thus resulting in a length change of the Fabry-Perot cavity, which later accordingly varies the light intensity of Fabry-Perot interference collected by the photodetector [12]. Therefore, the membrane deformation according to the specific PD-induced acoustic pressure determines the sensitivity of the whole fiber optic EFPI sensor system.

From the above description, as the most important sensing element, the membrane plays a vital role in the overall performance of the fiber optic EFPI sensor; e.g., the sensitivity and linear range, as well as the flatness of the central reflective region of the membrane which will influence the light intensity of Fabry-Perot interference [13,14].

### 2.1. IM Structure

**Figure 2:**
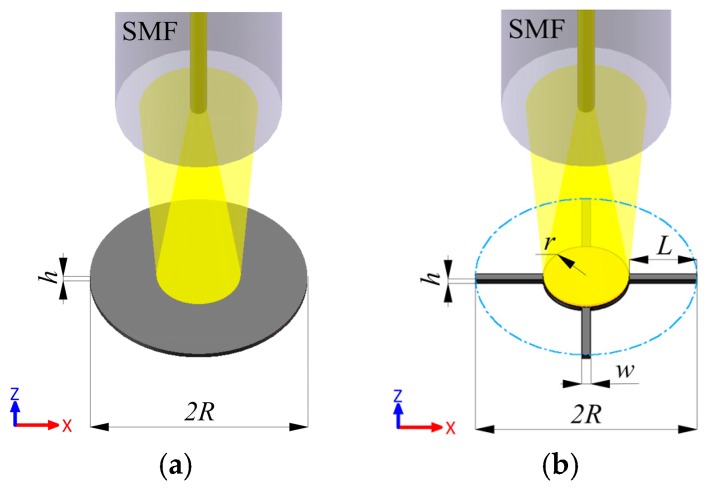
Schematic illustration of the membrane structure for detecting PDs: (**a**) intact membrane (IM); (**b**) beam-supported membrane (BSM).

In most cases, the IM structure, as shown in Figure 2a, is extensively adopted for sensing the acoustic signals, normally made of single crystal silicon with the membrane thickness of tens of micrometers [12,13,14,15,16,17]. Obviously, only two geometrical parameters are involved in designing the structure, i.e., film thickness *h* and radius *R*, both dependent on the fundamental frequency of membrane required for a certain magnitude of PDs. The fundamental frequency *f* of a circular membrane can be expressed by [18]:

(1) $$f=\frac{ah}{4\pi R^{2}}\sqrt{\frac{E}{3\rho (1-\mu^{2})}}=\alpha \frac{h}{R^{2}}$$

where *a* is the coefficient of vibration mode, *E*, *μ* and *ρ* is the elastic modulus, Poisson’s ratio, and density of the membrane material, respectively, and *α* is the equivalent coefficient. In general, the fundamental frequency of membrane is selected in the range of 70–180 kHz, considering the influence of the noise frequency distribution in normal transformers and the attenuation of the high-frequency ultrasonic waves in the oil [1,4,6,12,13,15].

When the acoustic pressure *p* is applied, the central deformation of membrane can be given by [18]:

(2) $$y(p)=\frac{3(1-\mu^{2})p}{16Eh^{3}}R^{4}=\beta \cdot p\frac{R^{4}}{h^{3}}$$

where *β* is also the equivalent coefficient. Usually, the greater the central deformation *y*(*p*) of membrane is, the higher the responsive sensitivity will be.

To design the membrane to get the maximal *y*(*p*), on the condition that the fundamental frequency *f* of membrane has already been decided by the PD type, *R* and *h* must increase or decrease at the same time according to the Equation (1). However, referring to the Equation (2), when *R* and *h* increase or decrease at the same time, the change of *y*(*p*) is still uncertain.

For the purpose of solving the above problem, by multiplying the Equations (1) and (2), there follows:

(3) $$f\cdot y(p)=\alpha \frac{h}{R^{2}}\cdot \beta \cdot p\frac{R^{4}}{h^{3}}=\alpha \beta \cdot p\frac{R^{2}}{h^{2}}=\alpha \beta \cdot p{\left(\frac{R}{h}\right)}^{2}$$

From the Equation (3), a conclusion can be drawn that when the fundamental frequency *f* of membrane is determined, the larger the ratio of *R/h* is, the greater central deformation *y*(*p*) of membrane will be under the same acoustic pressure *p*. In other words, finding the optimal value of *R* and *h* is equivalent to searching for the maximal ratio of *R/h*.

According to the Equation (1), in order to keep the fundamental frequency constant, the relationship between the radius change ∆*R* and the thickness change ∆*h* can be expressed by:

(4) $$\frac{h+\Delta h}{{\left(R+\Delta R\right)}^{2}}=\frac{h}{R^{2}}$$

which can be further described as:

(5) $$\Delta h=\frac{h[2R\Delta R+{\left(\Delta R\right)}^{2}]}{R^{2}}$$

Taking the Equation (5) into account, we can obtain the following expression:

(6) $$\frac{R+\Delta R}{h+\Delta h}=\frac{R}{h}\cdot \frac{R^{2}+R\Delta R}{R^{2}+2R\Delta R+{\left(\Delta R\right)}^{2}}<\frac{R}{h}$$

According to the Equation (6), the *R/h* ratio gradually decreases with *R* and *h* increasing. That is to say, the smaller *R* and *h* are, the greater *R/h* ratio will be. It then concludes that if the fundamental frequency has been determined, it needs the combination of thinner membrane and smaller radius, which will result in a larger deformation.

In general, the thickness *h* of IM structure is much larger than 20 μm due to the limitation of micromachining processes [12]. According to the above analysis, in order to greatly increase the sensitivity of the membrane, the Microelectromechanical Systems (MEMS) processing technology and Silicon-on-Insulator (SOI) wafers with a specifically defined device layer of the thickness much less than 20 μm are proposed to be utilized for manufacturing the membrane structure in this research.

### 2.2. BSM Structure

In order to further increase the sensitivity, BSM structure is proposed to replace the IM structure, as shown in Figure 2b. Intuitionally, there creates two extra design parameters of the BSM structure, length *L* and width *w* of the supporting beams, as the radius *r* of the central membrane is self-constraint (*r* = *R* − *L*). Moreover, the BSM structure can provide a larger linear range and a better flatness than its IM counterpart.

In addition, the BSM structure can provide a better resistance to the temperature change. For the normal IM structure, due to the totally sealed Fabry-Perot cavity, the temperature variation happening in the working environment of the EFPI sensor will create an unbalanced air pressure between the internal and external surface of the membrane structure, resulting in the deviation of the sensor’s sensitivity. Therefore, for practical applications, IM structure must drill an additional hole into the Fabry-Perot cavity to keep the pressure in balance [12,19]. This situation is now well eliminated for the BSM structure because of the existence of opening areas.

## 3. Results and Discussions

Both IM and BSM structures were comparably investigated to demonstrate the better sensitivity and higher design flexibility of the novel BSM structure. The fundamental frequency for the corresponding IM structure was assumed to be constant, thus the thickness *h* and radius *R* of the membrane were determined. The BSM structure with the same membrane dimension and thickness was then surveyed. Both structures were made of single crystal silicon. The peripheries of membrane structures were clamped. The smart-sizing method and multi-zone method were utilized to mesh the IM structures and BSM structures, respectively. The minimum side length was about 1.25 μm. Finally, the modal analysis, as well as the static structural analysis, was performed.

### 3.1. Frequency Response

Considering that the ultrasonic frequency of PDs is mainly about 150 kHz and a higher responsive frequency makes the EFPI sensor more reliable, the IM structure was designed with a resonant frequency around 180 kHz, which still possesses a relatively large responsive amplitude. To obtain the fundamental frequency of 180 kHz, the radius of circular IM structure is uniquely determined by the expected thickness *h*. The membrane thickness *h* of 5 μm, 10 μm, 15 μm, and 20 μm were set in this research for both structures. According to the Equation (1), the radius *R* of IM structure for each case was calculated and then verified by the simulations. Thus, for the BSM structure with the same dimension of *R*, when the length *L* of supporting beams is set, the radius *r* of the central membrane will be also known. Furthermore, the width *w* of supporting beams that can be possibly designed has a maximum value of $\sqrt{2}$ × *r*.

**Figure 3:**
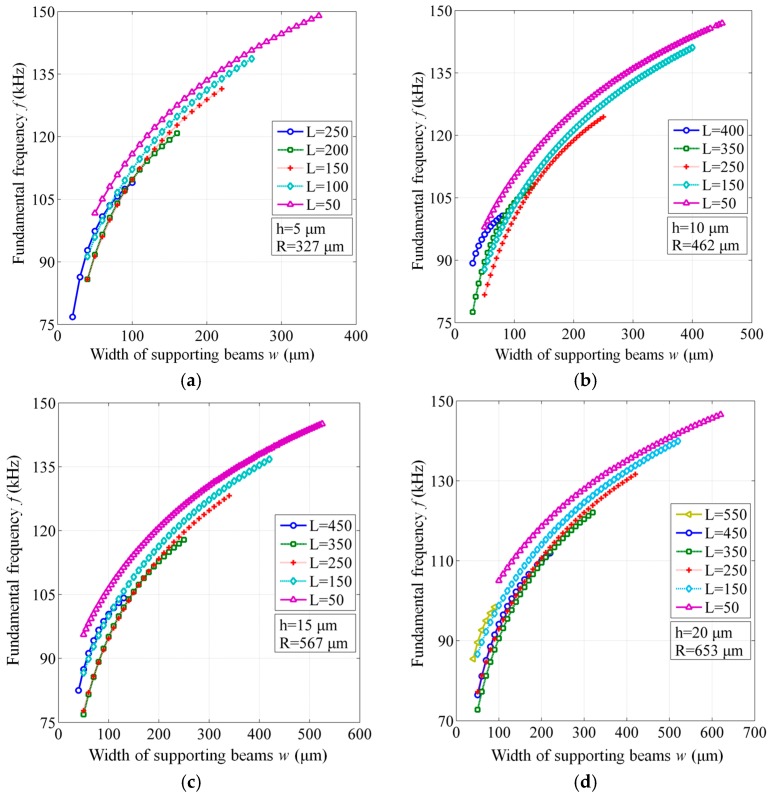
Fundamental frequency of BSM structures for different geometrical dimensions, compared to a constant value of 180 kHz for the IM structures. (**a**) Membrane thickness h = 5 μm and radius R = 327 μm; (**b**) h = 10 μm and R = 462 μm; (**c**) h = 15 μm and R = 567 μm; (**d**) h = 20 μm and R = 653 μm.

Figure 3 illustrates the fundamental frequency of BSM structures for different geometrical dimensions, while keeping that of all the IM structures 180 kHz. When the length *L* is decreased, a slight increase of the fundamental frequency can be observed. Comparatively, the width *w* has an obviously larger impact on the fundamental frequency. From the simulated results, the fundamental frequency realized by the BSM structures covers the range of 70 kHz to 150 kHz, showing a greatly enhanced design flexibility and a largely extended application potential compared to their IM counterparts.

### 3.2. Sensitivity

As mentioned in the Design Section, the sensitivity *S* for the two membrane structures can be defined by [1]:

(7) $$S=\frac{y(p)}{p}$$

where *p* represents the acoustic pressure applied on the membrane, the amplitude of which depends on how seriously the PDs reveal, as well as the distance and position of the PD source to the sensor. As indicated in the Equation (2), *y*(*p*) denotes the central deformation of membrane. For the IM structure, the sensitivity was thus calculated and confirmed by the finite element simulations. For the BSM structure, the finite element model was established to accurately express the complex geometry and derive the sensitivity for different cases with variant length *L* and width *w* of supporting beams. To simulate the membrane deformation under a PD-induced ultrasonic signal, both structures were loaded by a typical acoustic pressure ranging from 1 Pa to 10 kPa [20]. Then, the central displacement of the reflective membrane was recorded. By calculating the slope of the linear pressure-displacement line, the sensitivity *S* was derived.

**Figure 4:**
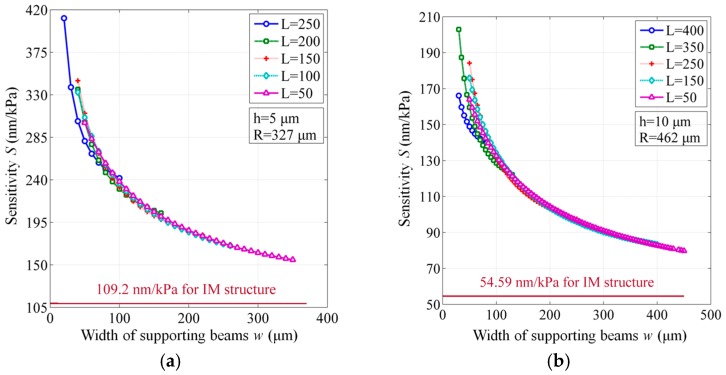

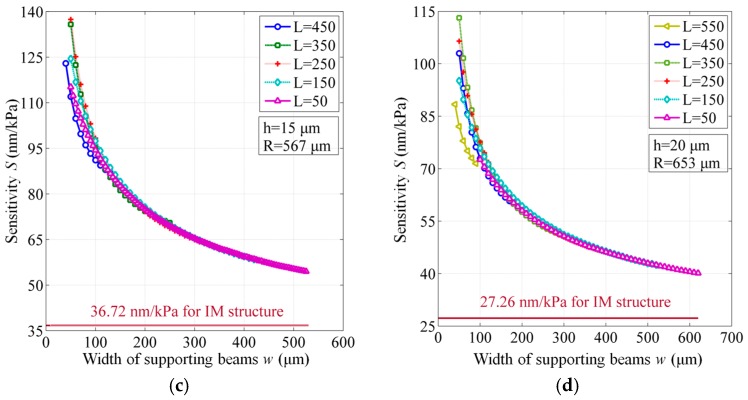
Sensitivity of BSM structures for different geometrical dimensions, compared to the constant values of IM structures. (**a**) Membrane thickness h = 5 μm and radius R = 327 μm; (**b**) h = 10 μm and R = 462 μm; (**c**) h = 15 μm and R = 567 μm; (**d**) h = 20 μm and R = 653 μm.

The achieved results for both membrane structures are listed in Table 1 and shown in Figure 4, from which we can see that all the BSM structures possess a much higher sensitivity than their IM counterparts. For some cases, the sensitivity is increased by almost four times, and we believe that the sensitivity can be further enhanced to a higher level via the design optimization.

In Figure 4, we can also observe that a rapid change of sensitivity appears especially when the width *w* is less than 100 μm. The narrower the width is, the larger the sensitivity would be. As a comparison, the length *L* shows a neglecting influence. On the other hand, as the membrane thickness increases, the sensitivity of both membranes drops substantially; this agrees well with the theoretical analysis for the IM structure in Section 2.1. Therefore, it suggests that the thickness of the membrane structure should be under well control during the fabrication of EFPI sensors, indicating one of the advantages of utilizing the SOI wafers in which cases the thickness of device layers for defining the membrane structures can be accurately customized as required.

### 3.3. Linear Range

**Figure 5:**
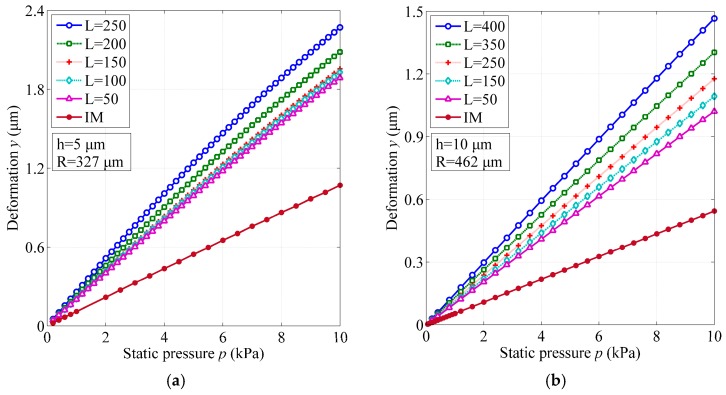

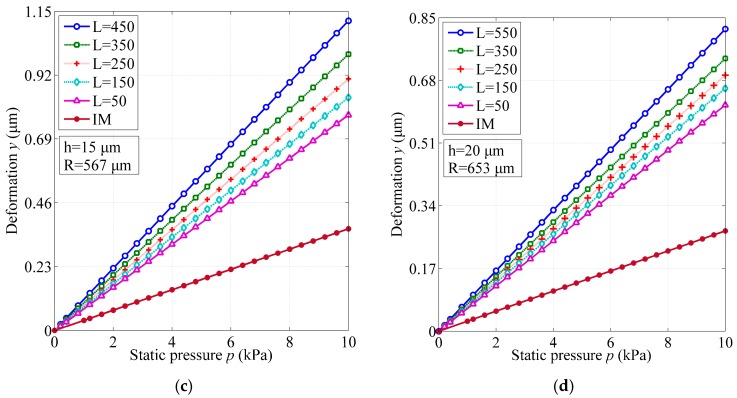
Linear responses of BSM structures and IM structures for different geometrical dimensions under typical acoustic pressures. (**a**) Membrane thickness h = 5 μm and radius R = 327 μm; (**b**) h = 10 μm and R = 462 μm; (**c**) h = 15 μm and R = 567 μm; (**d**) h = 20 μm and R = 653 μm.

According to the practical applications, the larger linear range of membrane deformation is beneficial to detect the PDs effectively. By the simulations under different acoustic pressures, the membrane deformation for both IM and BSM structures is given in Figure 5. Once finding the point of slope change, the linear ranges of BSM and IM structures can be obtained by dividing the maximum linear displacement by the membrane thickness, which are listed in Table 1. In Figure 5, we can obviously see that BSM structures present a larger deformation under a specific acoustic pressure, giving another evidence of the enhanced sensitivity for BSM structures. In addition, according to the results listed in Table 1, BSM structures own a larger linear range than IM structures for all the cases investigated in this research, although both structures seem to function well under the entire acoustic pressure range surveyed.

### 3.4. Flatness of Reflecting Area

**Figure 6:**
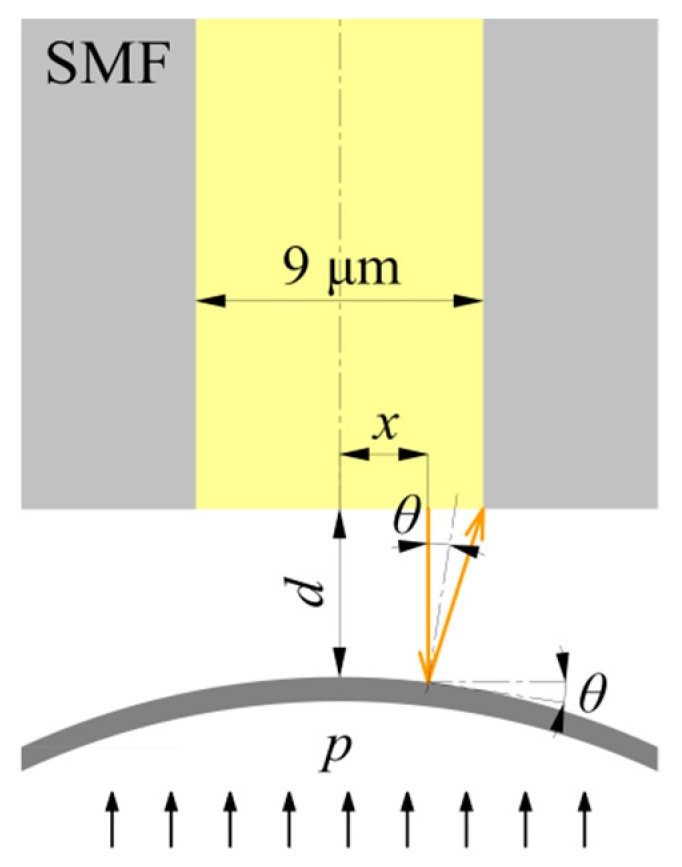
Schematic of the Fabry-Perot cavity between the end surface of fiber and membrane.

The membrane deformation caused by a PD acoustic pressure may lead to a non-parallel membrane surface, and correspondingly a non-uniform Fabry-Perot cavity. To obtain an enhanced signal-to-noise ratio (SNR) and a high optical throughput, the deformed membrane needs to have a good flatness of its central reflecting area. Figure 6 shows the illustrative diagram of how the membrane deformation affects the received light intensity through the SMF reflected from the membrane. The angle *θ* represents the degree of membrane deformation in the reflecting area. A smaller *θ* will ensure the sufficient light intensity received and high transmittance of Fabry-Perot cavity. With the assumption of normal incidence and a SMF of 9 μm core, a simplified calculation for the allowed maximum angle *θ_max_* is expressed as:

(8) $$\theta_{max}=\frac{1}{2}\arctan\frac{4.5-x}{d}$$

where *d* is the central cavity length. For example, taking *x* as 4.3 μm and *d* as 30 μm, *θ_max_* should be less than 0.19° to ensure that 95% of the reflected light re-enters into the SMF. Functionally speaking on this side, the required reflecting area on the membrane does not need to be so large. The complete design of the membrane structure for practical applications should take into account the fundamental frequency as required, as well as the assembly manner between the membrane structure and the SMF (referred to Figure 1b).

**Figure 7:**
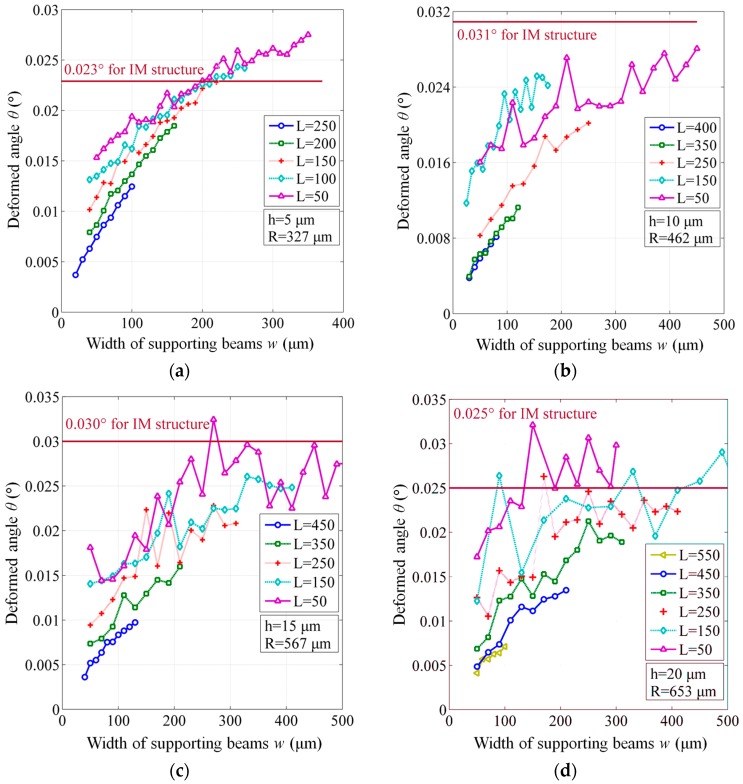
Flatness of BSM structures for different geometrical dimensions, compared to the constant values of IM structures. (**a**) Membrane thickness h = 5 μm and radius R = 327 μm; (**b**) h = 10 μm and R = 462 μm; (**c**) h = 15 μm and R = 567 μm; (**d**) h = 20 μm and R = 653 μm.

To study the flatness of both IM and BSM structures with different geometrical designs, the FEM simulations were performed under the largest deformation condition in the linearly elastic range, which is considered as 25% of the membrane thickness [12]. The results are listed in Table 1 and shown in Figure 7.

In Figure 7, most of BSM structures present a better flatness than IM structures. Although some fluctuations of the calculated angles exist (the reason of which is still not known), the main trend of all the curves can be distinctly observed. When the width *w* decreases, as well as the length *L* increases, the deformed angle *θ* of the membrane is reduced, indicating that a preferable flatness is realized. It is implied that the BSM structure can suppress the warping of membrane and maintain a parallel reflecting area. Therefore, the proposed BSM structures can raise the received light intensity into SMF and provide a higher SNR than IM structures.

## 4. Conclusions

A novel BSM structure for the fiber optic EFPI sensors with a better performance and an improved resistance to the temperature change was suggested, and its performances including natural frequency, sensitivity, linear range, and flatness were investigated in detail and compared with the IM structure. From the obtained results, we can see that the BSM structure provides extra geometrical parameters, covering a broader frequency range that can be possibly utilized for practical applications when the whole structural dimension and membrane thickness are kept constant, indicating a significantly enhanced design flexibility. In addition, the BSM structure not only increases the responsive sensitivity up to four times in comparison with the IM structure under a certain PD acoustic pressure, but also improves the linear range of the membrane deformation. Moreover, the BSM structure also presents a better flatness to make a higher SNR. The research work described in this paper is useful for the design of the EFPI sensor system that is capable of monitoring the weak signals of PD activities in power transformers as well as other acoustic events. Fabrication aspects and experimental demonstration of BSM structures will be discussed in the near future in order to prove its effectiveness. One more thing deserving mention is that the suggested BSM structure can not only suit for the intensity interrogation as the system illustrated in Figure 1, but can also apply to the detection system employing wavelength modulation.

## Figures and Tables

**Table 1:** Comparison of the realized performance for intact membrane (IM) and beam-supported membrane (BSM) structures.

Thickness *h* (μm)	Radius *R* (μm)	Frequency *f* (kHz)		Sensitivity *S* (nm/kPa)		Linear Range (%)		Flatness *θ* (°)	
		IM	BSM	IM	BSM	IM	BSM	IM	BSM
5	327	180	152.9–76.81	109.2	411.1–149.6	25	30	0.023	0.028–0.004
10	462		146.9–77.53	54.59	202.9–79.75	24	28	0.031	0.028–0.004
15	567		145.1–95.58	36.72	115.2–54.48	24	27	0.030	0.032–0.003
20	653		146.6–72.75	27.26	137.4–40.13	24	26	0.025	0.032–0.004

## References

[1] Yu B., Kim D.W., Deng J., Xiao H., Wang A. (2003). Fiber Fabry-Perot sensors for detection of partial discharges in power transformers. Appl. Opt..

[2] Wang Y., Gong S., Grzybowski S. (2011). Reliability evaluation method for oil–paper insulation in power transformers. Energies.

[3] Stone G.C. (2005). Partial discharge and electrical equipment insulation condition assessment. IEEE Trans. Dielect. Electr. Insul..

[4] Wang Q., Ma Z. (2013). Feedback-stabilized interrogation technique for optical Fabry–Perot acoustic sensor using a tunable fiber laser. Opt. Laser Technol..

[5] Wang M., Vandermaar A.J., Srivastava K.D. (2002). Review of condition assessment of power transformers in service. IEEE Electr. Insul. Mag..

[6] Yi W.H., Jin T., Xu G.H. (2016). Research progress on F-P interference-based fiber-optic sensors. Sensors.

[7] Lundgaard L.E. (1992). Partial discharge. XIII. Acoustic partial discharge detection-fundamental considerations. IEEE Electr. Insul. Mag..

[8] Lundgaard L.E. (1992). Partial discharge. XIV. Acoustic partial discharge detection-practical application. IEEE Electr. Insul. Mag..

[9] Dong B., Han M., Sun L., Wang J., Wang Y., Wang A. (2008). Sulfur hexafluoride-filled extrinsic Fabry–Perot interferometric fiber-optic sensors for partial discharge detection in transformers. IEEE Photon. Technol. Lett..

[10] Gander M.J., MacPherson W.N., Barton J.S., Reuben R.L., Jones J.D.C., Stevens R., Chana K.S., Anderson S.J., Jones T.V. (2003). Embedded micromachined fiber-optic Fabry-Perot pressure sensors in aerodynamics applications. IEEE Sens. J..

[11] Islam M.R., Ali M.M., Lai M.H., Lim K.S., Ahmad H. (2014). Chronology of Fabry-Perot interferometer fiber-optic sensors and their applications: a review. Sensors.

[12] Wang X., Li B., Xiao Z., Lee S.H., Roman H., Russo O.L., Chin K.K., Farmer K.R. (2005). An ultra-sensitive optical MEMS sensor for partial discharge detection. J. Micromech. Microeng..

[13] Li X., Shao Y., Yu Y., Zhang Y., Wei S.A. (2016). Highly sensitive fiber-optic Fabry–Perot interferometer based on internal reflection mirrors for refractive index measurement. Sensors.

[14] Shang Y., Ni Q., Ding D., Chen N., Wang T. (2015). Fabrication of optical fiber sensor based on double-layer SU-8 diaphragm and the partial discharge detection. Optoelectron. Lett..

[15] Song L., Wang Z., Wang A., Liu Y., Cooper K.L. (2006). Angular dependence of the frequency response of an extrinsic Fabry-Perot interferometric (EFPI) fiber acoustic sensor for partial discharge detection. J. Lightwave Technol..

[16] Wang X., Xu J., Zhu Y., Cooper K.L., Wang A. (2006). All-fused-silica miniature optical fiber tip pressure sensor. Opt. Lett..

[17] Zhu J., Wang M., Shen M., Chen L., Ni X. (2015). An optical fiber Fabry–Perot pressure sensor using a SU-8 Structure and angle polished fiber. IEEE Photon. Technol. Lett..

[18] Giovanni M.D. (1983). Flat and corrugated diaphragm design handbook. J. Mech. Work. Technol..

[19] Wang W., Yu Q., Jiang X. (2012). High sensitivity diaphragm-based extrinsic Fabry–Perot interferometric optical fiber underwater ultrasonic sensor. Adv. Mater. Rapid Commun..

[20] Posada-Roman J., Garcia-Souto J.A., Rubio-Serrano J. (2012). Fiber optic sensor for acoustic detection of partial discharges in oil-paper insulated electrical systems. Sensors.

